# Atypical Cause of Sepsis from Bilateral Iliopsoas Abscesses Seeded from Self-mutilation: A Case Report

**DOI:** 10.5811/cpcem.2020.5.47020

**Published:** 2020-07-07

**Authors:** Sam Langberg, Shayan Azizi

**Affiliations:** * University of Queensland School of Medicine, Ochsner Clinical School, Department of Emergency Medicine, New Orleans, Louisiana; † Ochsner Health System, Department of Emergency Medicine, Division of Emergency Ultrasound, New Orleans, Louisiana

**Keywords:** abscess, sepsis, iliopsoas abscess, self-mutilation

## Abstract

**Introduction:**

An iliopsoas abscess (IPA) is an abscess located adjacent to the iliopsoas and iliacus muscles. Although rare, their variable clinical presentations often lead to a delay in diagnosis.

**Case report:**

We present a case of sepsis secondary to multiple IPAs that was missed despite multiple healthcare encounters. The patient had no classical risk factors for an IPA, and the abscesses were found to be seeded via hematogenous spread from self-inflicted cutting.

**Conclusion:**

This case illustrates the importance of obtaining a complete history, including psychiatric screen, and performing a thorough examination when evaluating patients with low back pain to rule out overlooked sources of bacteremia.

## INTRODUCTION

An iliopsoas abscess (IPA) is an abscess located adjacent to the iliopsoas and iliacus muscles. An IPA can be classified as a primary abscess resulting from hematogenous spread of bacteria via the blood supply of the iliopsoas musculature, or a secondary abscess formed directly by adjacent infectious processes.^1^ It is a rare condition, with an annual incidence rate of 0.4 cases per 100,000 persons, but its true incidence is felt to be underdiagnosed and under-reported due to its vague presentation leading to delays in diagnosis and resulting increases in morbidity and mortality.^2^,^3^

The classic triad of fever, flank pain, and limitation of hip movement are only present in 30% of cases, and diagnosis remains a challenge because iliopsoas abscesses more commonly present with low-grade pyrexia, body aches, malaise, or non-specific abdominal or hip pain.^3^ Due to the complexity and variability in presentation, diagnosis typically depends on having a high clinical index of suspicion. If suspected, it is imperative to investigate in order to avoid mortality rates of 2.4% and 18.9% of primary and secondary IPAs, respectively.^1^ IPAs should be investigated with blood cultures and inflammatory markers, along with ruling out other sites of infection; however, laboratory studies are non-specific, and diagnosis ultimately relies on imaging with ultrasound, computed tomography (CT), or magnetic resonance imaging (MRI). Once diagnosed, treatment typically begins with empiric antibiotics, followed by percutaneous drainage.^1^,^4^

## CASE REPORT

A 19-year-old woman with a past medical history of schizoaffective disorder presented to the emergency department (ED) with a one-week history of left hip pain and chills. Pain was worsened with hip flexion and described as “searing” while sitting. She saw an orthopedic surgeon at the onset of the hip pain and was diagnosed with left hip bursitis. She was prescribed a course of prednisone and cyclobenzaprine. Despite use of the prednisone, she reported no improvement in symptoms. She also reported low-grade fever, poor appetite, polydipsia, fatigue, diarrhea, and insomnia. The patient had a history of cutting, but it had been over two months since she had last cut her forearms. This history was affirmed by the patient’s mother at bedside.

On initial ED presentation, the patient had a temperature of 36.8° Celsius, blood pressure of 115/59 millimeters of mercury (mm Hg), heart rate of 119 beats per minute (bpm), respiratory rate of 18 breaths per minute (breaths/min), and pulse oximetry measured 98% on room air. Physical examination was remarkable for left lumbar paraspinal tenderness. There was good range of motion of the left hip, with minimal pain on internal rotation and extension. There were multiple, self-inflicted, superficial lacerations in various stages of healing on her bilateral wrists. Serum laboratory studies were significant for a leukocytosis of 21.32 thousands per microliter (K/uL) (Reference [Ref]: 3.90–12.80 K/uL) and hyponatremia of 130 millimoles per liter (mmol/L) (Ref: 136–145 mmol/L), otherwise without significant abnormalities. A urinalysis was clear. Abdominal radiographs were normal. The leukocytosis and insomnia were attributed to the recent course of prednisone. She received a non-steroidal anti-inflammatory medication and was discharged home.

Three days later, she returned to her primary care doctor with worsening hip pain, fever, and lightheadedness. She was noted to be hypotensive with a blood pressure of 78/45 mm Hg. She was referred back to the ED. On her second ED visit, and fourth healthcare encounter since the onset of symptoms, she was noted to be febrile, with a temperature of 39.4° C, tachycardic, with a heart rate of 118 bpm, a blood pressure of 104/57 mm Hg, a respiratory rate of 18 breaths/min, and pulse oximetry measured 99% on room air. Examination revealed persistent left lumbar paraspinal tenderness with no midline spinal tenderness, and no rigidity to range of motion of hip. Laboratory studies revealed a worsening leukocytosis of 22.2 K/uL (Ref: 3.90–12.80 K/uL), an erythrocyte sedimentation rate (ESR) of >120 millimeters per hour (mm/hr) (Ref: 0–36 mm/hr), and a C-reactive protein (CRP) of 478.2 milligrams per liter (mg/L) (Ref: 0.0–8.2 mg/L).

**CPC-EM Capsule**

What do we already know about this clinical entity?

An iliopsoas abscess (IPA) is a rare pathologic entity with significant morbidity. The varied clinical presentations often lead to a delay in diagnosis.

What makes this presentation of disease reportable?

We present a case of sepsis secondary to IPAs seeded from self-mutilation that was missed on multiple health care encounters because the patient had none of the classic risk factors for IPAs.

What is the major learning point?

Providers should be suspicious for bacteremia or IPA in patients with a psychiatric history, or a history of self-mutilation, if presenting with fevers and pain in the back, abdomen, or hip.

How might this improve emergency medicine practice?

Given their variable presentation, providers may suspect and recognize an IPA earlier in a patient without classical risk factors for this disease process.

**Image 1:**
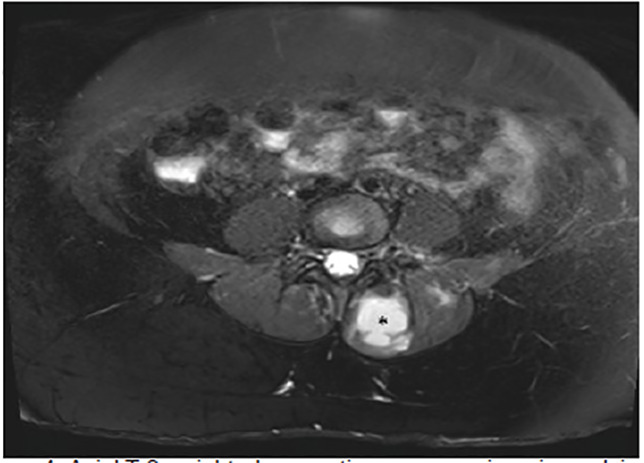
Axial T-2 weighted magnetic resonance imaging pelvis revealing a 3.4 x 3.2 x 7.9 centimeter abscess involving the left paraspinal muscle (*).

**Image 2:**
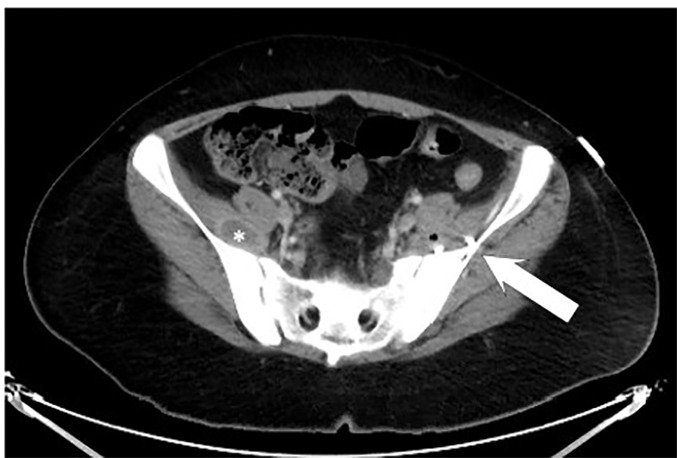
Computed tomography of the abdomen and pelvis with intravenous contrast revealing a 2.7 centimeter (cm) abscess of right iliacus muscle (*). This had increased in size from 1.6 cm on initial magnetic resonance imaging. Note pigtail catheter (arrow) from drainage of left-sided iliopsoas abscess.

Pelvic MRI was obtained, which revealed multiple bilateral IPAs (Image 1). A 3.6 x 8.5 x 10.6 centimeter (cm) abscess on her left iliacus involving her sacroiliac joint, a 3.4 x 3.2 x 7.9 cm abscess involving her left paraspinal muscle, and a right iliac abscess measuring 1.5 x 2.4 x 3.6 cm. She received broad-spectrum antibiotic coverage with vancomycin and piperacillin-tazobactam. Interventional radiology performed CT-guided aspiration with drain placement of the two larger, left-sided abscesses. Both blood and abscess cultures returned positive with methicillin-resistant *Staphylococcus aureus* (MRSA). Throughout her hospitalization, her white blood cell count continued to trend upward, and a CT revealed enlargement of the right iliacus abscess (Image 2). Interventional radiology subsequently performed a percutaneous aspiration of the abscess. She was discharged on hospital day 10 on parenteral vancomycin via a peripherally inserted central catheter line.

**Image 3:**
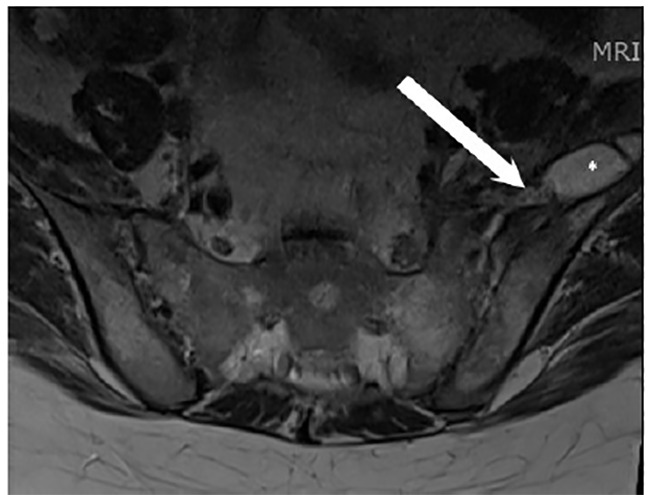
Axial T-2 weighted magnetic resonance imaging of lumbar spine after four weeks showing a recurrent abscess (*) measuring 3.2 x 1.3 centimeters. The lateral aspect of this collection extends beyond the field of view. The medial aspect of this collection appears continuous with the left sacroiliac joint (arrow), concerning for sacroilitis and possible involvement of the left iliac bone, although incompletely characterized on this study.

One month after discharge, the patient returned to the ED, reporting worsening back pain. Repeat MRI revealed persistence of left iliopsoas abscess measuring 3.2 x 1.3 cm, which now extended into the left iliac bone consistent with sacroiliitis/osteomyelitis (Image 3). She was evaluated by both interventional radiology and orthopedics consults who felt that the fluid collection was too small for aspiration or drain placement. Infectious diseases consult evaluated the patient and recommended a six-week course of intravenous (IV) daptomycin. As an outpatient, antibiotics were adjusted to oral doxycycline. Follow-up MRI two months after discharge revealed resolution of abscess with residual inflammatory edema.

## DISCUSSION

This case illustrates the high degree of morbidity from the misdiagnosis of a case of a primary IPA. This patient unfortunately experienced 93 days between her first ED visit to the resolution of her infection. Primary IPAs exist in the setting of bacteremia, whereas secondary IPAs may progress to bacteremia and sepsis. The high vascularity within the iliopsoas musculature, predisposes to the seeding of an abscess. This case may have been initially misdiagnosed because the patient had none of the classic risk factors for IPAs, such as IV drug use, immunodeficiencies, or inflammatory bowel disease where the risk of hematogenous seeding of bacteria is higher. The most common cause of a secondary IPA is Crohn’s disease, followed by appendicitis, diverticulitis, ulcerative colitis, osteomyelitis, neoplasm, disk infections, renal infections, and trauma.^5^ With none of the traditional risk factors for iliopsoas abscess, obtaining a focused psychiatric history to screen for self-harm could have given clues to the source of bacteremia and abscess formation.

*Staphylococcus aureus* is the most predominant organism cultured from IPAs.^1^ Other common pathogens cultured are species found on the skin or in the gastrointestinal tract, such as Staphylococcus, Streptococcus, *Escherichia coli*, and enterococcus, but often are polymicrobial.^1^ An IPA enlarges and creates mass effect on the adjacent iliopsoas and iliacus muscles. This will typically present with pain and swelling in the region irritating the hip extensors, leading to a limp and a positive psoas sign. However, the classic triad of decreased hip movement, flank pain, and fever may only be present in 30% of patients.^6^ There are cases in the literature of IPA presenting as exclusively fever and thigh pain.^1^,^7^

If there is suspicion for an IPA, serum laboratory studies such as an elevated white blood cell count, ESR, or CRP, are non-specific, and diagnosis requires imaging. IPAs may be identified on ultrasound, CT, or MRI. CT with contrast is frequently the initial imaging study given the feasibility and speed of CTs, but MRI is considered superior because of better discrimination of the soft tissues and the ability to visualize the abscess wall and surrounding structures without the need of an IV contrast medium.^8^

Even with treatment, primary and secondary IPAs have a mortality rate of 2.4% and 19%, respectively.^1^ Literature suggests that an untreated IPA may reach a mortality rate of up to 100%.^1^ Treatment and management of an IPA begins with empiric, broad-spectrum antibiotics for polymicrobial coverage, followed by percutaneous drainage.^1^,^4^ Open surgical drainage may be required for IPAs in the setting of Crohn’s disease because of the potential for abscesses to be connected via a fistula to the intestine, or in abscesses with multiple septae.^9^ Antibiotics are eventually tailored toward culture results. There is some literature suggesting that antibiotics alone may be adequate in abscesses smaller than 3 cm in greatest diameter.^10^

The literature describes a number of other unorthodox etiologies of IPAs. Tuberculosis has been known to seed as IPAs and may be suspected in patients with human immunodeficiency virus.^11^ In a case in India, the rare gram-negative organism, melioidosis, was cultured from an IPA.^12^ There have been cases of MRSA retroperitoneal infections attributed to infected skin lesions as a port of entry.^13^ However, on our review of the literature, this is the first case that describes an IPA as a result of self-mutilation.

## CONCLUSION

This case describes an atypical etiology of an IPA in a young patient with no classic risk factors, leading to a delay in diagnosis and treatment. Bacteremia is not exclusive to patients with a history of diabetes, immunosuppression, or IV drug use. In patients with a psychiatric history or a history of self-mutilation, providers should keep a level of suspicion for bacteremia or IPA in patients presenting with fevers and pain in the back, abdomen, or hip.
